# Sodium zirconium cyclosilicate treatment and rates of emergency interventions for hyperkalaemia: a propensity–score weighted case–control study

**DOI:** 10.1093/ckj/sfae313

**Published:** 2024-10-21

**Authors:** William R Marshall, Gabriel A Curran, Jamie P Traynor, Keith A Gillis, Patrick B Mark, Jennifer S Lees

**Affiliations:** School of Cardiovascular and Metabolic Health, University of Glasgow, Glasgow, UK; School of Cardiovascular and Metabolic Health, University of Glasgow, Glasgow, UK; Glasgow Renal and Transplant Unit, NHS Greater Glasgow and Clyde, Glasgow, UK; School of Cardiovascular and Metabolic Health, University of Glasgow, Glasgow, UK; School of Cardiovascular and Metabolic Health, University of Glasgow, Glasgow, UK; School of Cardiovascular and Metabolic Health, University of Glasgow, Glasgow, UK

**Keywords:** acute kidney injury, chronic kidney disease, dialysis, hyperkalaemia, potassium binders

## Abstract

**Background:**

Sodium zirconium cyclosilicate (SZC) reduces serum potassium in patients with chronic hyperkalaemia in clinical trials, but its role in the emergency treatment of hyperkalaemia is unproven. We hypothesized that SZC use for emergent hyperkalaemia would be associated with a reduction in rates of emergency interventions for hyperkalaemia.

**Methods:**

This was a single-centre, propensity score–weighted case–control study of patients admitted with hyperkalaemia to a specialist renal centre. We randomly selected 250 patients admitted between April 2021 and September 2022 (post-SZC era) with a potassium level ≥5.5 mmol/l treated with at least one ≥10 g dose of SZC (treatment group). We randomly selected a comparator group of 250 patients admitted between January 2018 and December 2019 (pre-SZC era) with a potassium level ≥5.5 mmol/l (control group). Baseline demographic and clinical characteristics were recorded and used as covariates for propensity scoring and inverse probability treatment weighting (IPTW). Our primary outcome measure, rates of emergency haemodialysis (HD), was tested using unadjusted models and multivariable logistic regression models on unweighted data in addition to unadjusted models on weighted data. We also reviewed rates of emergency temporary central venous access as a secondary outcome.

**Results:**

A total of 59% were male, the mean age was 67 years (standard deviation 14) and 149 (30%) were receiving maintenance dialysis. IPTW achieved satisfactory balance of covariates between the treatment and control groups. In the treatment group, patients were 77% less likely to need emergency HD {odds ratio [OR] 0.23 [confidence interval (CI) 0.17–0.31]}. This result was consistent following analysis of weighted and unweighted data. Similarly, patients treated with SZC were 73% less likely to require emergency temporary central venous access [OR 0.27 (CI 0.20–0.36)].

**Conclusion:**

SZC was associated with a significant reduction in the rates of emergency HD and emergency temporary central venous access in patients admitted to a specialized renal centre with emergent hyperkalaemia.

## Graphical Abstract

**Graphical Abstract:**
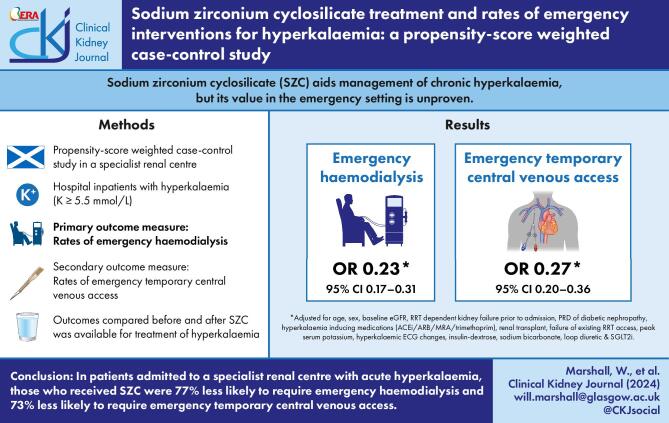

## INTRODUCTION

Hyperkalaemia represents a medical emergency when an increase in serum potassium occurs acutely and/or is associated with electrocardiographic (ECG) changes. Emergent hyperkalaemia can occur in the context of acute kidney injury (AKI) due to several mechanisms: direct injury to tubular potassium-secreting cells, a significant reduction in the glomerular filtration rate or oligoanuria with a significant decrease in distal tubule salt and water delivery. It also commonly occurs in chronic kidney disease (CKD), particularly when the estimated glomerular filtration rate (eGFR) decreases to <15–20 ml/min and there is limited capacity to further increase potassium excretion. The early focus of emergency hyperkalaemia treatment is on stabilizing the myocardium and redistributing serum potassium intracellularly. Definitive management consists of inducing potassium loss from the body, either by gastrointestinal (GI) tract potassium binders and/or emergency haemodialysis (HD), often via an emergency temporary central venous catheter (CVC) [1].

Traditional cation exchange–based resins are limited in their ability to decrease serum potassium reliably and significantly [2, 3]. Furthermore, adverse effects are common and can be life-threatening; bowel necrosis is a well-described complication [4–6]. Emergency HD via an emergency temporary CVC is a highly effective way of removing serum potassium, but is not always available in a timely fashion, nor is it always clinically appropriate.

Sodium zirconium cyclosilicate (SZC) is a newer GI tract binder that works throughout its intestinal transit, exchanging potassium ions for sodium and hydrogen ions [7, 8]. The onset of action is rapid, with a reported mean reduction in serum potassium of 0.2 mmol/l within the first hour following administration. SZC also has a more favourable side-effect profile compared with traditional cation exchange–based resins, which contain sorbitol and often cause significant GI side effects [9].

The efficacy of SZC has been demonstrated in studies evaluating the non-emergency treatment of hyperkalaemia, when serum potassium does not climb acutely and/or is not associated with ECG changes [10, 11]. In light of these studies, SZC was first approved for use in Scotland in September 2020 by the Scottish Medicines Consortium (SMC) with a licence restricting its use to CKD and heart failure patients to maintain therapeutic doses of renin–angiotensin–aldosterone system inhibitors [12]. In addition, UK guidelines made recommendations for SZC use in the emergency treatment of hyperkalaemia, despite a dearth of supportive evidence in this setting; the National Institute for Health and Care Excellence recommended SZC alongside standard care for the emergency treatment of hyperkalaemia in September 2019, with a starting dose of 10 g every 8 hours [13]. The UK Kidney Association subsequently made the same recommendations in July 2020, with SZC featuring again in their updated guidelines in October 2023 [14, 15]. SZC use in the emergency setting was considered off-licence in Scotland from September 2020 onwards before the SMC expanded the licence to include the emergency treatment of hyperkalaemia in November 2022 [16].

In this study, we report our experiences with the emergency treatment of hyperkalaemia before and after the availability of SZC in a specialist renal centre. We hypothesized that SZC use would be associated with a reduction in rates of emergency HD and emergency temporary CVC use.

## MATERIALS AND METHODS

### Study population and setting

This retrospective study was conducted in the Glasgow Renal and Transplant Unit in NHS Greater Glasgow and Clyde. The unit covers a population of 1.6 million, including 500 prevalent HD patients.

We conducted a propensity score–weighted case–control study of patients admitted to our unit with hyperkalaemia, comparing patients managed before (January 2018–December 2019) and after (April 2021–September 2022) SZC was available for treatment of hyperkalaemia in the inpatient setting.

We obtained Caldicott Guardian approval for use of patient-identifiable data in this study. The data used for this study were collected by regular members of the medical team and are routinely recorded on our unit in day-to-day care, thus ethics approval and explicit patient consent were not required.

### Data sources

All patients admitted during the periods of interest were identified from the electronic patient record (EPR; VitalPulse UK). The EPR holds all demographic and clinical information of patients encountered by our service.

We extracted age, sex, baseline eGFR (based on serum creatinine; Chronic Kidney Disease Epidemiology Collaboration equation 2009 [17]), primary renal diagnosis (PRD) of diabetic nephropathy, renal replacement therapy (RRT)-dependent kidney failure prior to admission [peritoneal dialysis (PD) in addition to HD], use of hyperkalaemia-inducing medications and peak serum potassium for each patient. Peak serum potassium (hospital-drawn sample processed on the same day in the on-site biochemistry lab) was defined as the highest serum potassium (mmol/l) for which the patient received hyperkalaemia treatment during the inpatient stay.

Hyperkalaemia-inducing medications considered were angiotensin-converting enzyme inhibitors (ACEis), angiotensin II receptor blockers (ARBs), mineralocorticoid receptor antagonists (MRAs), trimethoprim, non-steroidal anti-inflammatory drugs (NSAIDs), amiloride and beta blockers. Those patients on more than one hyperkalaemia-inducing medication were considered as one exposure. We also recorded the total number of patients who had all their hyperkalaemia-inducing medications stopped while undergoing treatment.

The type of RRT access was recorded in patients who had usable access but had not yet started RRT. Patients with failure of an existing RRT access preventing regular HD sessions were included. We also recorded the administration of other medical therapies for the emergency treatment of hyperkalaemia or those that are known to reduce the incidence of hyperkalaemia: insulin-dextrose infusions, sodium bicarbonate, loop diuretics, sodium–glucose co-transporter 2 inhibitors (SGLT2is), thiazide diuretics and nebulized beta agonists. Receiving one or more doses (regardless of the dose) of these medications for treatment of hyperkalaemia was considered an exposure; those patients admitted on these therapies who continued them during the inpatient stay were also counted as an exposure.

We stratified peak serum potassium levels into mild (5.5–6.0 mmol/l without ECG changes), moderate (5.5–6.0 mmol/l with ECG changes or 6.1–6.5 mmol/l without ECG changes) and severe (6.1–6.5 mmol/l with ECG changes or >6.5 mmol/l). Peaked T waves, wide PR interval, prolonged QRS duration, loss of P waves, sinusoidal waves, bradyarrhythmia or tachyarrhythmia were all considered hyperkalaemic ECG changes [18].

Clinical and demographic details, particularly relating to PRD, concurrent use of hyperkalaemia-inducing medications, failure of existing RRT access, ECG changes, administration of calcium resonium, statins, insulin–dextrose infusions, sodium bicarbonate, loop diuretics, SGLT2is, thiazide diuretics and nebulized beta agonists were checked by the primary investigator (W.R.M.) in a detailed case note review.

Any emergency HD session and/or emergency temporary CVC performed within our unit is recorded in the EPR as part of unit protocol. If performed in a setting outside our renal unit (e.g. in intensive care), these procedures are not reliably recorded in the EPR and were therefore not considered for the purposes of this study.

All patients treated with insulin–dextrose infusion(s) who went on to require emergency HD had the duration of time from the first insulin–dextrose infusion to the first emergency HD session recorded. In those treated with SZC, we also reviewed the serum potassium before the first SZC dose and then following the final dose. In those patients treated with SZC who required emergency HD, the serum potassium after the final dose was taken as the last one available prior to commencing the first emergency HD session. Patients in the control group requiring emergency HD also had their last serum potassium prior to their first HD session recorded.

Patients treated with SZC during the period of interest were identified by a research query in our hospital electronic prescribing record (HEPMA; WellSky, Bristol, UK). HEPMA provided exact dosages and frequencies and allowed us to ensure prescribed doses were administered to patients.

### Participants/population

All patients admitted to the NHS Greater Glasgow and Clyde renal unit between April 2021 and September 2022 (post-SZC era) with a peak serum potassium ≥5.5 mmol/l were considered for the treatment group. Those admitted with a peak serum potassium <5.5 mmol/l were excluded. Any patient with a peak serum potassium ≥5.5 mmol/l treated with one or more ≥10-g dose of SZC was deemed suitable for the treatment group; one dose was deemed sufficient for treatment group allocation given existing studies demonstrating the significant impact of SZC from the very first dose. Those patients treated with subtherapeutic (<10 g) doses of SZC were excluded. Patiromer was not routinely available for the treatment of hyperkalaemia in our unit during the periods of interest. It was therefore administered to a very small number of patients and thus they were excluded from our study. We also excluded patients admitted in the post-SZC era who received calcium resonium.

All patients admitted to the NHS Greater Glasgow & Clyde renal unit between January 2018 and December 2019 (pre-SZC era) with a peak serum potassium ≥5.5 mmol/l were considered for the control group. Those admitted with a peak serum potassium <5.5 mmol/l were excluded.

While conventional hyperkalaemia treatments may have been given prior to admission to our renal unit and did not preclude involvement in our study, all patients dialysed in emergency outside of our unit in the pre- and post-SZC eras were excluded from our analysis. In our centre, covering a large geographical area in the West of Scotland, the vast majority (>95%) of acute HD is conducted directly within our unit, with external HD (in intensive care or coronary care units) conducted only in exceptional circumstances. We are likely to have captured almost all situations where acute HD was required for emergent hyperkalaemia.

Patients who had a peak serum potassium ≥5.5 mmol/l that was successfully medically treated to <5.5 mmol/l but required emergency HD for other indications such as uraemia or fluid overload were excluded from this study.

To assess the representativeness of the selected patients in the treatment and control groups, we reviewed the baseline characteristics of all remaining patients admitted during the periods of interest. The ‘untreated’ group included all those admitted in the post-SZC era with a peak serum potassium ≥5.5 mmol/l who did not receive any SZC, patiromer or calcium resonium. The ‘unselected’ group included patients admitted with a peak serum potassium ≥5.5 mmol/l who were not randomly selected for the control group from the pre-SZC era.

We used calcium resonium treatment as a positive control exposure and statin treatment as a negative control exposure [19]. Calcium resonium was the only other traditional cation exchange–based resin binder available in our unit at the time of this study, which (if effective) would be expected to produce a positive effect similar to SZC in reducing the likelihood of the outcomes of interest. Statin exposure was selected as a negative control exposure, as this was a commonly co-prescribed medication among patients in our study and would not be expected to have any significant bearing on the likelihood of receiving SZC treatment nor of reaching the outcomes of interest. In this way, the negative control was used to explore and account for unmeasured confounding in the study.

### Outcomes

The outcome measures were retrieved from the EPR:

- Primary outcome: Emergency HD, defined as any HD session(s) occurring during the inpatient admission to the unit, either for a previously non-dialysis-dependent patient or an extra HD session outside of the regular regimen of a dialysis-dependent patient. Ultrafiltration-only sessions for management of fluid overload were not included as an emergency HD outcome.
- Secondary outcome: Emergency temporary CVC, defined as a non-tunnelled CVC (either internal jugular, clavicular or femoral) inserted during the admission to the unit. This was included as an outcome to review rates of emergency temporary CVC for patients with existing (failing) dialysis access as well as in those without established HD access.

### Statistical analysis

Baseline characteristics were recorded and summarized as mean [standard deviation (SD)], median [interquartile range (IQR)] or number (percentage) and compared with *t*-test, Kruskal–Wallis test or χ^2^ test as appropriate. We initially ran an unadjusted logistic regression model (Model 1) on unweighted data to determine the likelihood of emergency HD and emergency temporary CVC in the treatment and control groups, generating odds ratios (ORs) with 95% confidence intervals (CIs) according to SZC exposure, calcium resonium exposure (positive control) and statin exposure (negative control). Sequential multivariable logistic regression models were subsequently run on unweighted data to adjust for potential explanatory variables using the Wald test: Model 2: age and sex; Model 3: Model 2 plus baseline eGFR and RRT-dependent kidney failure prior to admission; Model 4: Model 3 plus renal transplant recipient, PRD of diabetic nephropathy, hyperkalaemia-inducing medications, failure of existing RRT access, peak serum potassium, hyperkalaemic ECG changes, insulin–dextrose infusions, sodium bicarbonate, loop diuretics, SGLT2is, thiazide diuretics and nebulized beta agonists.

We then used propensity scoring (PS) and inverse probability treatment weighting (IPTW) to identify and correct imbalances in covariates between the treatment and control groups. We reweighted participants to reach a satisfactory balance in covariates between the two groups, defined as a standardized mean difference <±0.05 and a variance ratio <2. Covariates were selected based on biological plausibility of an association with the outcome(s) and/or treatment allocation and were included regardless of the potential for collinearity with other covariates [20, 21]. We ran unadjusted logistic regression models for the outcomes of interest on the reweighted populations. All statistical analyses were performed using SPSS (IBM, Armonk, NY, USA) and R statistical software version 4.0.4 within RStudio for Mac version 2023.02.1 (R Foundation for Statistical Computing, Vienna, Austria) using the tidyverse, ggplot2, dbpylr, tableone, cobalt, survival and broom packages.

## RESULTS

### Demographic and clinical characteristics of patients

**Figure 1:**
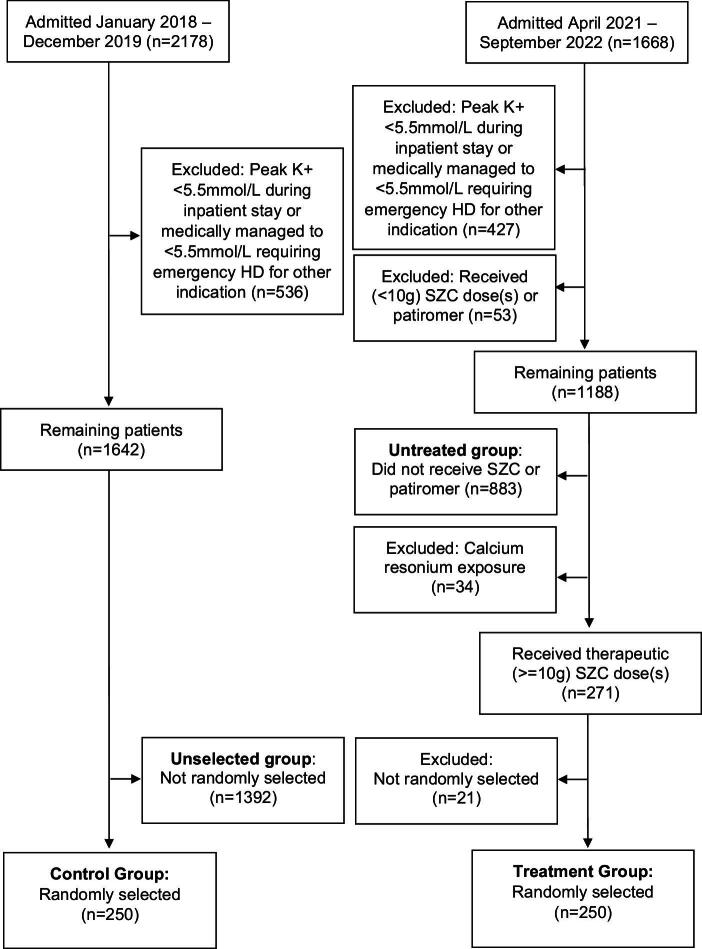
Consort flow diagram showing selection of patients for the study.

A total of 1668 patients were admitted in the post-SZC era; 1241 had a peak serum potassium during admission of ≥5.5 mmol/l while 427 were excluded due to a peak serum potassium <5.5 mmol/l or because they were medically managed to this level but required emergency HD for another indication. Of the remaining 1241 patients, 883 did not receive any SZC and formed the untreated group; 47 were administered a sub-therapeutic SZC dose(s) (<10 g) and a further 6 received patiromer, meaning 53 were excluded. A total of 34 were treated with calcium resonium during the hospital admission and were therefore excluded from the treatment and untreated groups; these were used as positive control outcomes. A total of 271 patients received a therapeutic (≥10 g) dose(s) of SZC, of which 250 were randomly selected to form the treatment group (Fig. 1). Of the 250 selected patients, 79 were treated with a statin and were used as negative control outcomes.

A total of 2178 patients were admitted in the pre-SZC era, of which 1642 patients had a peak serum potassium ≥5.5 mmol/l; 536 were excluded due to a peak serum potassium <5.5 mmol/l or because they were medically managed to this value but required emergency HD for another indication. A random number generator selected 250 patients of the 1642 for analysis as the control group (Fig. 1). The remaining 1392 patients made up the unselected group. A total of 88 patients in the control group were treated with calcium resonium during the hospital admission and were used as positive control outcomes; 76 were treated with a statin and were used as negative control outcomes.

**Figure 2:**
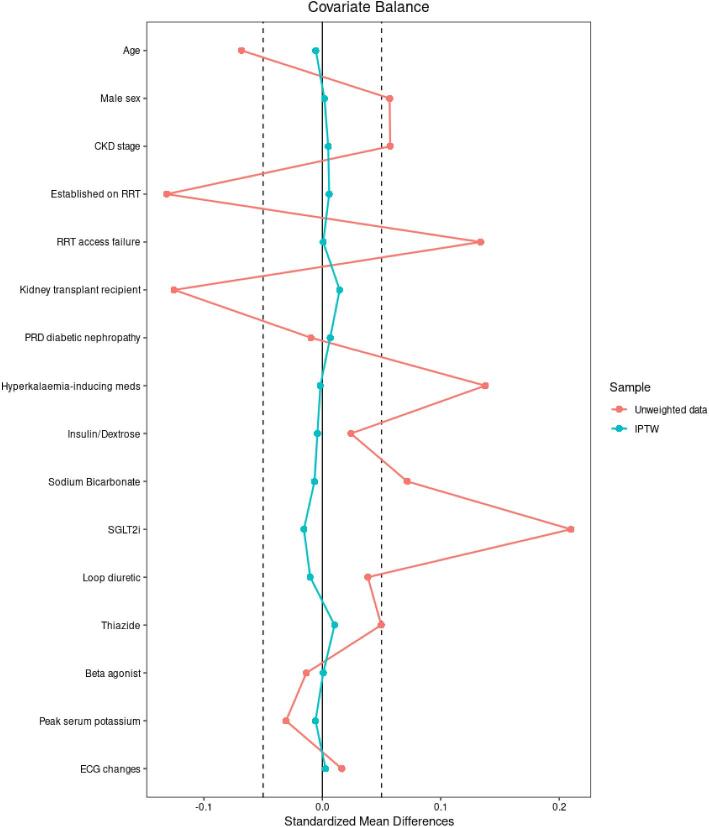
Covariate balance plot demonstrating standardised mean differences (SMD) across covariates in unweighted and weighted data. Threshold SMD <±0.05 (dashed line).

The baseline characteristics of patients in the treatment and control groups appeared generally well matched (Table 1). However, after calculating PS and IPTW in the unweighted data, the standardized mean differences (SMD) of several covariates were demonstrated to be above the accepted threshold for significance of <±0.05 (Supplementary Table S1). Following weighting, all covariates were well balanced between the two groups and the SMD fell below the significance limit (Fig. 2).

**Table 1:** Baseline characteristics of unmatched patients in the control and treatment groups.

Characteristics	Control	Treatment	*P*-value
Patients, *n*	250	250	
Age (years), mean (SD)	67.04 (14.05)	66.06 (14.67)	0.446
Male, *n* (%)	143 (57.2)	150 (60.0)	0.586
eGFR (ml/min/1.73 m^2^), *n* (%)			0.015
≥ 50	76 (30.4)	63 (25.2)	
≥30–<50	20 (8.0)	45 (18.0)	
≥15–<30	48 (19.2)	45 (18.0)	
<15	106 (42.4)	97 (38.8)	
RRT dependent prior to admission, *n* (%)	82 (32.8)	67 (26.8)	0.171
Dialysis access, *n* (%)			0.059
Arteriovenous fistula	45 (18.0)	37 (14.8)	
Arteriovenous graft	10 (4.0)	10 (4.0)	
Peritoneal dialysis catheter	2 (0.8)	7 (2.8)	
Tunnelled central venous catheter	32 (12.8)	17 (6.8)	
Failure of existing RRT access, *n* (%)	20 (8.0)	30 (12.0)	0.103
Renal transplant recipient, *n* (%)	34 (13.6)	24 (9.6)	0.209
PRD of diabetic nephropathy, *n* (%)	57 (22.8)	56 (22.4)	1.000
Total patients on hyperkalaemia-inducing meds, *n* (%)	88 (28.8)	96 (35.2)	0.150
ACEi/ARB	34	47	
Trimethoprim	9	6	
MRA	11	14	
NSAID	19	17	
Amiloride	2	0	
Beta blocker	23	20	
Peak potassium (mmol/l), mean (SD)	6.31 (0.81)	6.28(0.65)	0.614
Mild hyperkalaemia, *n* (%)	97 (38.8)	84 (33.6)	0.142
Moderate hyperkalaemia, *n* (%)	38 (15.2)	58 (23.2)	0.189
ECG changes, *n* (%)	7 (18.4)	15 (25.9)	0.171
Severe hyperkalaemia, *n* (%)	115 (46.0)	108 (43.2)	0.536
ECG changes, *n* (%)	87 (75.7)	84 (77.8)	0.753
Total patients with ECG changes, *n* (%)	94 (37.6)	99 (39.6)	0.547
Insulin–dextrose infusion, *n* (%)	137 (54.8)	140 (56.0)	0.724
Sodium bicarbonate, *n* (%)	65 (26.0)	73 (29.2)	0.484
Loop diuretic, *n* (%)	54 (21.6)	58 (23.2)	0.751
SGLT2i, *n* (%)	8 (3.2)	20 (8.0)	0.032
Nebulized beta agonist, *n* (%)	25 (10.0)	24 (9.6)	0.981
Thiazide diuretic, *n* (%)	14 (5.6)	17 (6.8)	0.613

The baseline characteristics of patients in the untreated and unselected groups appeared well balanced in relation to each other (Table 2).

**Table 2:** Baseline characteristics of patients in unselected and untreated groups.

	Unselected	Untreated
Characteristics	group	group
Patients, *n*	1392	883
Age (years), mean (SD)	66.1 (14.7)	65.6 (13.6)
Male, *n* (%)	779 (60.0)	512 (58.0)
eGFR (ml/min/1.73 m^2^), *n* (%)		
≥ 50	530 (38.1)	316 (35.8)
≥30–<50	264 (19.0)	135 (15.3)
≥15–<30	153 (11)	150 (17.0)
<15	445 (32.0)	282 (31.9)
RRT dependent prior to admission, *n* (%)	370 (26.6)	254 (28.7)
Renal transplant recipient, *n* (%)	135 (9.7)	122 (13.8)
PRD of diabetic nephropathy, *n* (%)	293 (21.0)	224 (25.4)
Peak potassium (mmol/l), mean (SD)	6.24 (0.52)	6.27 (0.41)

The median number of doses of SZC per patient treated was 8 (IQR 4–11), with a median duration of treatment of 4 days (IQR 2–8). The mean serum potassium in the treatment group prior to receiving any SZC was 6.53 mmol/l (SD 0.41); in those who did not undergo emergency HD, the mean serum potassium after completion of SZC was 5.12 mmol/l (SD 0.37). In those treated with SZC who required emergency HD, the mean serum potassium before emergency HD was 6.81 mmol/l (SD 0.35), which was comparable to the pre-emergency HD control group mean of 6.77 mmol/l (SD 0.31).

In patients receiving insulin–dextrose infusions for the treatment of hyperkalaemia, the median time from the first infusion to the first emergency HD session in the control group was 2.9 days (IQR 2.1–5.7) compared with 3.1 days (IQR 2.4–6.3) in the treatment group.

### Outcomes

In the control group, 45.2% (113/250) underwent emergency HD compared with 18.0% (45/250) in the treatment group (Table 3). We reviewed the characteristics of patients who required emergency HD in the treatment group and control group versus those who did not require emergency HD (Table 4).

**Table 3:** Total number of primary outcome measures (emergency HD) and secondary outcome measures (emergency temporary CVC) in treatment versus control groups.

Outcome measure	Control	Treatment	*P*-value
Emergency HD, *n* (%)	113 (45.2)	45 (18.0)	<0.001
Emergency temporary CVC, *n* (%)	98 (39.2)	43 (17.2)	<0.001

**Table 4:** Characteristics of patients in the treatment and control groups who required emergency HD versus those who did not.

Characteristics	Control, HD	Treatment, HD	Control, no HD	Treatment, no HD
Patients, *n*	113	45	137	205
Age (years), mean (SD)	65.12 (14.63)	64.68(14.72)	67.24 (13.95)	66.78 (14.17)
Male, *n* (%)	63 (55.8)	26 (57.8)	80 (58.4)	124 (60.5)
eGFR (ml/min/1.73 m^2^), *n* (%)				
≥50	31 (27.4)	14 (31.1)	45 (32.8)	49 (23.9)
≥30–<50	6 (5.3)	8 (17.8)	14 (10.2)	37(18.1)
≥15–<30	16 (14.2)	5 (11.1)	32 (23.4)	40 (19.5)
<15	51 (45.1)	18 (40.0)	55 (40.1)	79 (38.5)
RRT dependent prior to admission, *n* (%)	24 (21.2)	13 (28.9)	58 (42.3)	54 (26.4)
Failure of existing RRT access, *n* (%)	11 (9.7)	5 (11.1)	9 (6.6)	25 (12.2)
Renal transplant recipient, *n* (%)	4 (3.5)	2 (4.4)	30 (21.9)	22 (10.7)
PRD of diabetic nephropathy, *n* (%)	20 (17.7)	8 (17.8)	37 (27.0)	48 (23.4)
Patients on hyperkalaemia-inducing meds, *n* (%)	26 (23.0)	19 (42.2)	56 (40.9)	66 (37.6)
Peak potassium (mmol/l), mean (SD)	6.29 (0.57)	6.29 (0.52)	6.31 (0.49)	6.28 (0.54)
Mild hyperkalaemia, *n* (%)	28 (24.8)	14 (31.1)	69 (50.3)	70 (34.1)
Moderate hyperkalaemia, *n* (%)	12 (10.7)	7 (15.6)	26 (19.0)	51 (24.9)
ECG changes, *n* (%)	4 (33.3)	3 (42.9)	3 (11.5)	12 (23.5)
Severe hyperkalaemia, *n* (%)	73 (64.6)	24 (53.3)	42 (30.7)	84 (41.0)
ECG changes, *n* (%)	60 (82.2)	20 (83.3)	27 (64.3)	64 (76.2)
Total patients with ECG changes, *n* (%)	64 (56.6)	23 (51.1)	30 (21.9)	76 (37.0)
Insulin-dextrose infusion, *n* (%)	73 (64.6)	30 (66.7)	64 (46.7)	110 (53.7)
Sodium bicarbonate, *n* (%)	41 (36.3)	16 (35.6)	24 (17.5)	57 (27.8)
Loop diuretics, *n* (%)	24 (21.2)	13 (28.9)	30 (21.9)	45 (22.0)
SGLT2i, *n* (%)	3 (2.7)	5 (11.1)	5 (3.6)	15 (7.3)
Nebulised beta agonists, *n* (%)	7 (6.2)	5 (11.1)	18 (13.1)	19 (9.3)
Thiazide diuretics, *n* (%)	8 (7.1)	3 (6.7)	6 (4.4)	14 (6.8)

In the control group, 39.2% (98/250) required an emergency temporary CVC compared with 17.2% (43/250) in the treatment group. Among patients who were not already receiving maintenance RRT, 39.9% (67/168) in the control group required temporary CVC access versus 14.2% (26/183) in the treatment group. There was no significant difference (*P* = .37) in the rates of hyperkalaemia-inducing medications being stopped in the control group [82/88 (93.2%)] and the treatment group [85/96 (88.5%)].

**Figure 3:**
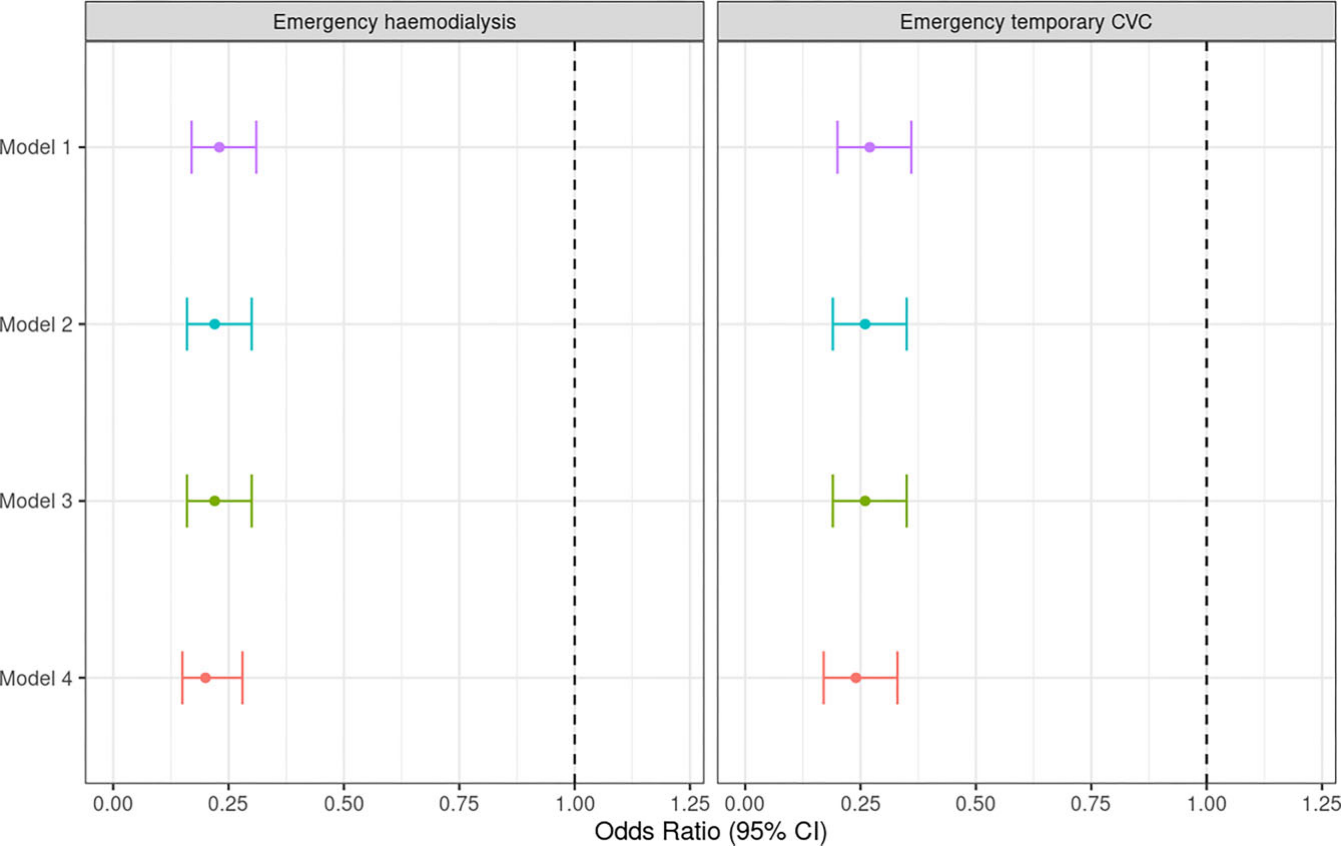
Forest plots demonstrating likelihood of primary outcome (emergency HD - left) and secondary outcome (emergency temporary CVC - right) in patients treated with SZC after adjustment for potential explanatory variables on unweighted data using models 1-4. Dashed line corresponds to no treatment effect.

In unadjusted logistic regression models on unweighted data, patients treated with SZC were 77% less likely to require emergency HD [OR 0.23 (CI 0.15–0.35)] and 72% less likely to require an emergency temporary CVC [OR 0.28 (CI 0.18–0.43)]. These differences were consistent after sequential adjustment for potential explanatory variables using Models 2, 3 and 4 (Fig. 3).

In unadjusted logistic regression models on weighted data, patients treated with SZC were 77% less likely to need emergency HD [OR 0.23 (CI 0.17–0.31)] and 73% less likely to require an emergency temporary CVC [OR 0.27 (CI 0.20–0.36)].

In logistic regression models on weighted data, the treatment effect of SZC was similar in men and women (emergency HD for sex–SZC interaction *P* = .06; emergency temporary CVC for sex–SZC interaction *P* = .59).

There was no significant difference in the rates of emergency HD and emergency temporary CVC between our positive control (calcium resonium; *P* = .97 and .97, respectively) and negative control exposures (statin therapy; *P* = .54 and .96, respectively).

## DISCUSSION

We believe this to be the first study exploring the real-world utility of SZC in reducing emergency procedures associated with hyperkalaemia. Randomized controlled trials in this setting are challenging to undertake and the only trials to date evaluating SZC use in this context have used surrogate outcome measures to demonstrate effect, including the reduction in serum potassium and number of doses of insulin–dextrose infusions [22, 23]. Our data show that SZC is associated with a substantial reduction in emergency HD and emergency temporary CVCs in patients with hyperkalaemia in the inpatient setting.

We believe our findings have significant implications for the acute treatment of hyperkalaemia. Clinicians must often assess risk, including the likelihood of requiring emergency HD, when deciding to admit (or not admit) to an acute site providing emergency HD. This can be particularly difficult in the early phases of acute hyperkalaemic kidney injury where trajectory is uncertain. Clinicians may be reassured that the likelihood of requiring emergency HD may be substantially reduced with SZC treatment; this may improve confidence in managing patients with AKI and hyperkalaemia outside an acute site providing HD.

Similarly, patients with advanced, non-RRT-dependent CKD (who may have planned elective creation of a dialysis access) or RRT-dependent patients with failure of an existing access may ordinarily require admission for an emergency temporary CVC and emergency HD in the event of hyperkalaemia. In our study we identified several patients who, in the absence of any other indications for emergency HD such as uraemia or fluid overload, were managed safely with SZC until a long-term dialysis access could be (re-)established.

The insertion of an emergency temporary CVC itself is not without risks; these may include pneumothorax, cardiac arrhythmia and line sepsis [24]. Emergency HD also carries its own share of morbidity and mortality in addition to that which comes with hyperkalaemic renal failure [25, 26]. Furthermore, established treatments for acute hyperkalaemia—such as insulin–dextrose infusions—have been shown to be associated with significant morbidity, particularly with repeated doses in which iatrogenic hypoglycaemia is associated with increased rates of death [27, 28]. Treatment of acute hyperkalaemia is often required in the out-of-hours setting and patients may be comorbid, frail or delirious, which can compound the baseline risks of emergency treatment for hyperkalaemia. SZC presents clinicians with a conservative treatment option that is efficacious and has a favourable safety profile.

While we did not assess cost-effectiveness in our study, it is important to note the current daily cost is ≈£30 for a therapeutic dose of SZC (10 g every 8 hours) [29]. SZC makes no considerable time or operator requirements on healthcare staff. Furthermore, the onset of action is reliably ≈1 hour and there are no known significant adverse events. Emergency temporary CVC and emergency HD carry the large unavoidable costs of equipment, staff and operator dependence, maintenance and disposal [30, 31]. Adverse events are often serious and can add significantly to these costs. The challenge ahead is ensuring SZC use is widespread in the inpatient setting, hyperkalaemia protocols need updating to incorporate SZC, medical staff (including those in non-renal departments) need educating on the use of SZC and therapeutic doses need to be readily available from medication cupboards.

We acknowledge the limitations of this study. The first and major limitation of this study is that it is a retrospective single-centre study. We cannot account completely for unmeasured confounders within this population, nor do the identified associations prove causality.

However, a randomized controlled trial or prospective study with real-world utility in this area is unlikely to be carried out; this case–control study with PS and IPTW seems the next best alternative. We used IPTW to adjust for imbalances for patient characteristics as a single covariate despite a relatively small number of events, but IPTW can only account for measured (and not unmeasured) confounders. Furthermore, IPTW is sensitive to misspecification of the PS model (where the chosen model does not accurately capture the relationship between covariates, including interaction effects, and treatment assignment), which may lead to biased effect estimates. Despite these limitations, we found similar estimates of effect using both multivariable regression and IPTW, which does strengthen our findings, although there remain substantial limitations in this retrospective approach.

Although we sampled from the population to ensure feasibility of a detailed case note review, random selection of patients for the treatment and control groups may result in selection bias and does not guarantee representativeness from the sample population. Similarly, the effect estimates may have been biased by the population sampling. Our analysis of the untreated and unselected largely supports similarities between the unselected and selected populations, although there are discrepancies in the breakdown of CKD stages between patients in the selected and unselected populations. It is unclear whether this represents a true difference (e.g. a COVID-era effect relating to which patients were admitted to our renal unit) or whether this represents categorization bias due to the way we recorded eGFR; recording eGFR as an absolute value and analysing this as a continuous variable would have given better insights into this.

Patients in the treatment group were also more likely to be on a SGLT2is, an expected finding given the broadening of clinical indications for prescription of SGLT2is beyond diabetes, and including CKD. SGLT2is have subsequently been shown to reduce the risk of hyperkalaemia when used in patients with non-dialysis-dependent CKD [32, 33]. Given the treatment and control groups also spanned time points before and after the onset of the COVID-19 pandemic, medical decision-making may have been influenced by other non-medical factors. The availability of SZC may also have changed attitudes towards the emergency treatment of hyperkalaemia, with clinicians more committed to aggressive medical therapy than in the pre-SZC era, where cation exchange resins were an unfavourable cornerstone of treatment. However, the conclusions were robust after reweighting the data to account for measured confounders and testing with positive and negative control exposures.

Our study design was not appropriate to evaluate the impact of SZC on other important aspects of emergency hyperkalaemia management, such as rebound hyperkalaemia following initial treatment. We also have no data on the safety and side effects of SZC in our patients; SZC carries a large sodium load (800 mg per 10-g dose) that may be problematic in a population at significant risk of fluid overload. Furthermore, despite calcium resonium failing to demonstrate any benefit in reducing emergency temporary CVCs and emergency HD when used as a positive control exposure in our populations, this is not a replacement for a prospective study comparing SZC directly with traditional cation exchange resins. Future work by Cañas *et al.* [34] is likely to provide more information in these areas.

In view of these limitations, we recognize that SZC appears to be an effective adjuvant treatment alongside other cornerstone treatments for emergent hyperkalaemia, including insulin–dextrose infusions and sodium bicarbonate. Our findings do not change the practice in severe or life-threatening hyperkalaemia, where the treatment of choice is still emergency HD to achieve a reliable and sustained reduction in the serum potassium.

## CONCLUSION

In a heterogeneous group of patients admitted to a specialist renal centre with acute hyperkalaemia, SZC use was associated with a signification reduction in the need for our primary outcome measure, emergency HD. SZC was also associated with a significant reduction in the rates of emergency temporary CVCs. This may potentially alter the paradigm of a common medical emergency, which has traditionally required resource-intensive treatment associated with significant morbidity and mortality. While these results require further prospective confirmation, our data support better renal outcomes after administration of SZC in inpatients with acute hyperkalaemia.

## Supplementary Material

sfae313_Supplemental_File

## Data Availability

The data that support the findings of this study are available from the corresponding author upon reasonable request.
