# Supplementary material for: Sodium zirconium cyclosilicate treatment and rates of emergency interventions for hyperkalaemia: a propensity–score weighted case–control study
Source: Clin Kidney J. 2024 Oct 21;17(11):sfae313. doi: 10.1093/ckj/sfae313 (PMC11635375; doi:10.1093/ckj/sfae313)
Supplement: sfae313_Supplemental_File [file sfae313_Supplemental_File.pdf]

Supplementary table 1 – Standardised mean difference (SMD) of covariates in unweighted and weighted data. All covariates met accepted level of significance (SMD <±0.05) following re weighting

	SMD in unweighted data	SMD in weighted data
Age	- 0.07078	- 0.00603
Male sex	0.04045	0.00087
Baseline eGFR	0.05492	0.00683
RRT dependent prior to admission	- 0.11478	0.00299
Failure of existing RRT access	0.13609	- 0.00255
Kidney transplant recipient	- 0.10000	0.01686
PRD of diabetic nephropathy	- 0.01906	0.00491
Hyperkalaemia-inducing medications	0.16215	- 0.00413
Peak serum potassium	- 0.30806	- 0.00733
Hyperkalaemic ECG changes	0.03303	0.00029
Insulin-dextrose infusion	0.03223	- 0.01161
Sodium bicarbonate	0.09040	- 0.00090
Loop diuretic	0.04877	- 0.00825
SGLT2i	0.22385	- 0.02040
Thiazide	0.05016	0.00491
Nebulised beta agonist	-0.03510	0.00311
